# A sensitive and rapid electrochemical biosensor for sEV-miRNA detection based on domino-type localized catalytic hairpin assembly

**DOI:** 10.1186/s12951-023-02092-x

**Published:** 2023-09-09

**Authors:** Wenbin Li, Wen Wang, Shihua Luo, Siting Chen, Tingting Ji, Ningcen Li, Weilun Pan, Xiaohe Zhang, Xiaojing Wang, Ke Li, Ye Zhang, Xiaohui Yan

**Affiliations:** 1 Laboratory Medicine Center, Department of Laboratory Medicine, Nanfang Hospital, School of Basic Medical Sciences, Southern Medical University, Guangzhou, 510515 People’s Republic of China; 2 Guangdong Engineering and Technology Research Center for Rapid Diagnostic Biosensors, Nanfang Hospital, Southern Medical University, Guangzhou, 510515 People’s Republic of China; 3 Medical Laboratory of the Third Affiliated Hospital of Shenzhen University, Shenzhen, 518001 People’s Republic of China; 4 Center for Clinical Laboratory Diagnosis and Research, The Affiliated Hospital of Youjiang Medical University for Nationalities, Baise, 533000 Guangxi People’s Republic of China

**Keywords:** DNA nanowire, Catalyzed hairpin assembly, sEV-miRNAs, Early cancer diagnostics

## Abstract

Small extracellular–vesicule-associated microRNA (sEV-miRNA) is an important biomarker for cancer diagnosis. However, rapid and sensitive detection of low-abundance sEV-miRNA in clinical samples is challenging. Herein, a simple electrochemical biosensor that uses a DNA nanowire to localize catalytic hairpin assembly (CHA), also called domino-type localized catalytic hairpin assembly (DT-LCHA), has been proposed for sEV-miRNA1246 detection. The DT-LCHA offers triple amplification, (i). CHA system was localized in DNA nanowire, which shorten the distance between hairpin substrate, inducing the high collision efficiency of H1 and H2 and domino effect. Then, larger numbers of CHAs were triggered, capture probe bind DT-LCHA by exposed c sites. (ii) The DNA nanowire can load large number of electroactive substance RuHex as amplified electrochemical signal tags. (iii) multiple DT-LCHA was carried by the DNA nanowire, only one CHA was triggered, the DNA nanowire was trapped by the capture probe, which greatly improve the detection sensitivity, especially when the target concentration is extremely low. Owing to the triple signal amplification in this strategy, sEV-miRNA at a concentration of as low as 24.55 aM can be detected in 20 min with good specificity. The accuracy of the measurements was also confirmed using reverse transcription quantitative polymerase chain reaction. Furthermore, the platform showed good performance in discriminating healthy donors from patients with early gastric cancer (area under the curve [AUC]: 0.96) and was equally able to discriminate between benign gastric tumors and early cancers (AUC: 0.77). Thus, the platform has substantial potential in biosensing and clinical diagnosis.

**Graphical Abstract:**

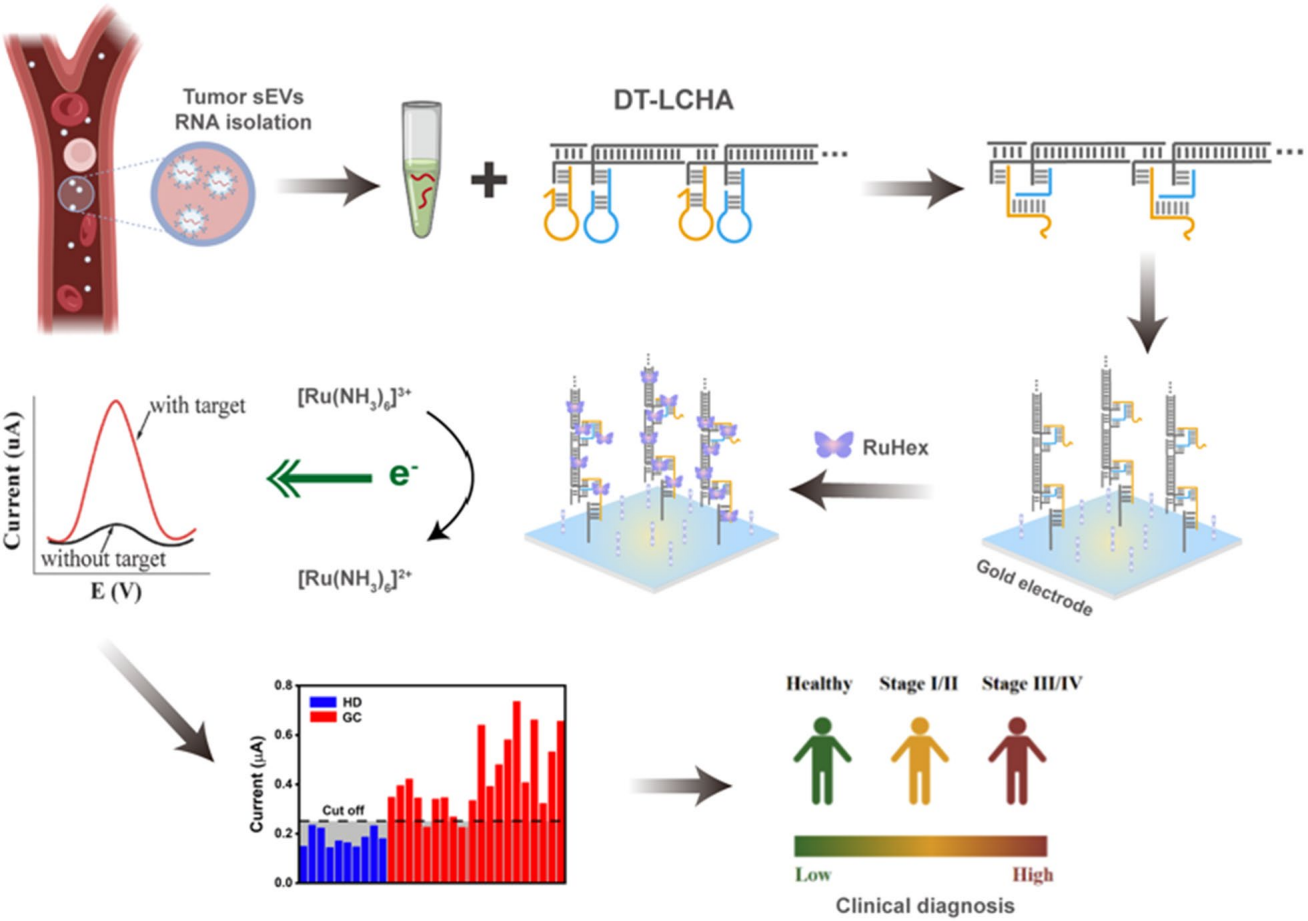

**Supplementary Information:**

The online version contains supplementary material available at 10.1186/s12951-023-02092-x.

## Introduction

Small extracellular vesicles (sEVs) are lipid nanoparticles secreted by eukaryotic cells and range in size from 20 to 200 nm [1, 2]. These vesicles contain various biological macromolecules, such as proteins, nucleic acids, and lipids. They play an important role in intercellular communication, as well as in diverse physiological and pathological processes [3, 4]. sEVs have an abundance of noncoding ribonucleic acids (RNAs), including circular RNA (CircRNA), microRNA (miRNA), and long noncoding RNA (lncRNA), participating in the regulation of target cell signaling and are closely associated with the progression of various diseases [5, 6]. Among these RNAs, sEV-miRNA has been found to be differentially expressed in patients with early-stage cancer compared with healthy people and has a higher stability than circulating miRNA in the circulatory system owing to the protection offered by the phospholipid bilayer [7]. Thus, sEV-miRNA is considered a promising cancer biomarker.

Reverse transcription quantitative polymerase chain reaction (RT-qPCR) is the current primary method for sensitive detection of sEV-miRNA [8, 9]. However, it has certain limitations, such as high reagent usage, expensive temperature-controlled equipment, and cumbersome operation [10, 11]. Researchers have developed multiple strategies for sEV-miRNA detection, including nanomaterial-based detection strategies [12–17], enzymatic amplification [18–21], and deoxyribonucleic acid (DNA) circuits [22–26]. Among these, DNA circuits such as catalytic hairpin assembly (CHA) and entropy-driven amplification circuits have emerged as promising alternatives to RT-qPCR because of their high specificity, ease of design, and superior stability [27–29]. However, DNA circuits pose challenges in clinical use owing to their low sensitivity and poor efficiency.

To address these challenges, localized DNA circuits (LDCs) have been proposed [30, 31]. LDCs use carriers with which DNA circuits are conjunct, thereby reducing the distance between reaction units and facilitating a higher regional concentration of the substrate [32, 33]. For example, DNA cube localized CHA (LCHA), used by Xu et al. for exosome miRNA detection, displayed higher response efficiency [34]. However, because DNA cubes have limited binding sites and can only localize a small number of CHA circuits, the sensitivity of this approach is considerably low. Bai et al. designed an LDC, a single-strand nanowire-based LCHA, to detect miRNA by localizing a high concentration of hairpin substrates on different nanowires [35]. Although this strategy provides high sensitivity, it requires the assembly of multiple nanowires, resulting in complex and time-consuming preparation. Therefore, developing an LDC with simple preparation and high sensitivity is essential.

Herein, we have designed a domino-type localized CHA (DT-LCHA)–based electrochemical platform for sensitive detection of sEV-miRNA. Compared with other LDCs, the DT-LCHA affords easy assembly (loading CHA hairpins on the single-stranded DNA nanowire) and offers triple amplification, (i). CHA system was localized in DNA nanowire, which shorten the distance between hairpin substrate, inducing the high collision efficiency of H1 and H2 and domino effect. Then, larger numbers of CHAs were triggered, capture probe bind DT-LCHA by exposed c sites. (ii) The DNA nanowire can load large number of electroactive substance RuHex as amplified electrochemical signal tags. (iii) multiple DT-LCHA was carried by the DNA nanowire, only one CHA was triggered, the DNA nanowire was trapped by the capture probe, which greatly improve the detection sensitivity, especially when the target concentration is extremely low. This platform has also been successfully applied to the clinical diagnosis of early-stage gastric cancer. Thus, the DT-LCHA-based electrochemical platform holds substantial potential for sensitive and clinically relevant detection of sEV-miRNA to support cancer diagnosis.

## Experiment section

### Materials and instruments

Oligos used in this platform were synthesized and purified by high performance liquid chromatography (HPLC) by Shanghai Sangon Co. The detailed sequences are shown in Additional file 1: Table S1. 6-mercapto-1-hexanol (MCH) and RuHex electrochemical molecules were purchased from Sigma-Aldrich (St. Louis, MO, USA), while cells and fetal bovine serum (FBS) were provided by Procell (Wuhan, China). phosphate buffer saline (PBS), Roswell Park Memorial Institute 1640 (RPMI-1640) and Dulbecco’s modified eagle medium (DMEM) were provided by Gbico, Thermo Fisher Scientific (MA, USA). Trizol was used for total RNA extraction and was provided by Fdbio science (Hangzhou, China). TNAK buffer was composed of 125 mM of NaCl, 20 of mM Tris powder and 20 of mM KCl, adjusting to pH = 7.5 by using HCl.

Electrochemical analysis was conducted using the CHI660E platform (Shanghai Chenhua Instruments Inc.) for differential pulse voltammetry (DPV), impedance spectroscopy (EIS) and square wave voltammetry (SWV). The electrochemical analysis platform construct was shown in Additional file 1: Fig S1. Agarose gel electrophoresis was imaged and analyzed using a Syngene electrophoresis analyzer (Syngene, England). Relative quantification was performed using RT-qPCR on a Roche light cycle 480.

### Preparation of DT-LCHA

To ensure proper spatial structure, all DNA sequences used in this study were heated at the temputure of 95 °C lasting 5 min using a PCR instrument, followed by slow cooling to room temperature. L1, L2, and Trigger were mixed in a 2:2:1 ratio and appropriately diluted with TNAK buffer. The mixture was incubated overnight at room temperature to form nanowires, followed by the cross-linking of H1 and H2 into the nanowires for 30 min to form the DT-LCHA system.

### Electrochemical biosensor preparation

At first, the 2 mm diameter gold disc electrode was polished with alumina powder for 6 min. The electrode was then cleaned using an ultrasonic cleaning machine with distilled water until no turbidity was observed in the ultrasound. The bare gold electrode was cleaned 3 times with piranha solution (H_2_O_2_: H_2_SO_4_ = 1:3 v/v) for 10 min each. The gold surface was then repeatedly rinsed with Tris–HCl to remove impurities. The capture probe was dropwise added to the gold electrode surface and stored overnight at 4 °C for the gold-thiol reaction.

### Electrochemical analysis for DT-LCHA

The gold electrode coated with the capture probe was closed with MCH for 1 h, with the capture probe kept as perpendicular to the gold plane as possible. Afterwards, DT-LCHA was mixed with the sample to be tested and added dropwise to the gold electrode surface for 20 min. Subsequently, the platinum wire electrode was employed as the counter electrode, the saturated calomel electrode served as the reference electrode, and the modified gold electrode functioned as the test electrode during the DPV analysis in 10 mM of Tris–HCl buffer from − 0.5 to 0.2 V with an amplitude of 0.05 V, a pulse width of 0.05 s, a sample width of 0.025 s, a pluse period of 0.2 s. EIS analysis was meticulously conducted within a 0.4 M KCl medium containing 0.5 mM [Fe(CN)_6_]^3−/4−^. The parameters were set as follows: an initial potential (E initial) of 0.195 V, high frequency (10000 Hz), low frequency (0.01 Hz), and an amplitude of 0.005 V. Subsequently, SWV analysis was executed in a 0.4 M KCl solution incorporating 0.5 mM [Fe(CN)_6_]^3−/4−^. The voltage sweep ranged from 0.5 to − 0.1 V, with an amplitude of 0.025 V and a frequency of 10 Hz.

### Fluorescence measurements for DT-LCHA

The functionalized fluorescent molecule FAM is aligned at the junction of the stem ring on H1 with the black hole quencher BHQ1, which is triggered by miR-1246 to release fluorescence. Fluorescence was recorded every 1 min for 30 min using a fluorescence spectrometer (PerkinElmer LS-55, USA) at excitation light 488 nm and emission light 515 nm.

### Isolation of EV and total RNA

Cell supernatant and plasma extractions were performed according to the guidelines established in the minimal information for studies of EVs 2018 (MISEV 2018) consensus statement. Briefly, cells were starved at 70% density for 12 h and incubated with exosome-depleted serum for 36–48 h. The cell supernatant was passed through a 0.22 μm filter to remove large particles. The sEVs were then pelleted by ultracentrifugation at 170,000 g for 1.5 h. For plasma sEV extraction, 1 mL of plasma was mixed with 1 mL PBS, then use ultracentrifugation at 170,000 g lasting 1.5 h to collect the sEV precipitate. The sEVs were washed twice with 75% ethanol and resuspended in DEPC water to obtain total RNA.

## Results and discussion

### Principle underlying the DT-LCHA platform

**Scheme 1.**
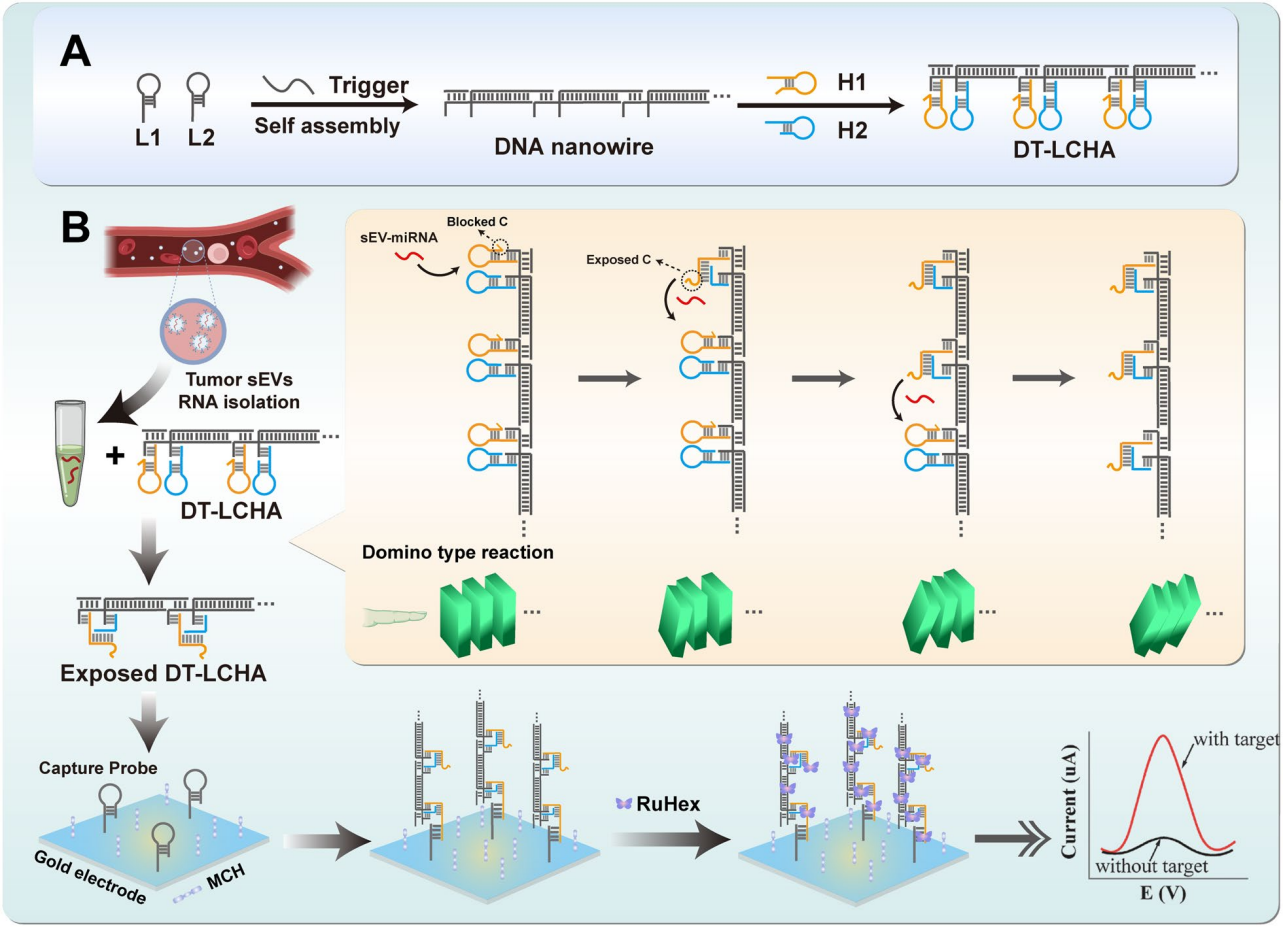
Schematic diagram of electrochemical detection platform based on DT-LCHA. **A** The construction process of DT-LCHA. **B** Detection process of sEV-miRNA with DT-LCHA electrochemical sensor

The principle underlying the functioning of the DT-LCHA platform is illustrated in Scheme 1. Because of the high expression of sEV-miR-1246 in patients with gastric tumors, it was used as a model molecule to verify this concept [36, 37]. As the design of DT-LCHA in Additional file 1: Fig S2, nanowires with sticky ends are formed by the hybridization of L1 and L2 in the presence of the trigger strand, and the CHA hairpins are loaded onto the sticky ends by complementary base pairing. Once the target sEV-miRNA1246 is added to the reaction mixture, the hairpin H1 opens into straight-stranded H1, which then leads to the opening of H2, exposing the blocked c site and releasing the sEV-miR-1246; the released sEV-miR-1246 then triggers the next CHA system by fallowing the direction of nanowire. In this manner, a domino-type triggering of CHA circuits occurs, ensuring easy activation of numerous CHA circuits. Next, DT-LCHA can be captured by the pairing of the exposed c site with the capture probe. The electrochemical signal molecule RuHex is then electrostatically bound to the DT-LCHA complexes, generating an amplified signal for detecting sEV-miRNA.

### Validation and characterization of the DT-LCHA platform

After the CHA system was designed in the manner shown in Additional file 1: Fig S2, its secondary structure and free energy were analyzed using the Nucleic Acid Package (NUPACK) software (Additional file 1: Fig S3) [38]. The double-stranded sequence obtained after hybridization of H1 and H2 has a free energy of − 42.32 kcal/mol, which is lower than the individual free energies of H1 and H2. Therefore, the H1/H2 hybrid is stable and the CHA reaction is irreversible. The exposed c site of the H1/H2 complex further pairs with the capture probe; because the hybrid structure thus formed has a lower free energy (− 56.20 kcal/mol), it is stable. These results indicate that when CHA is triggered by the target sEV-miRNA, it can form a stable double-stranded complex, which is subsequently captured by the capture probe.

**Fig. 1:**
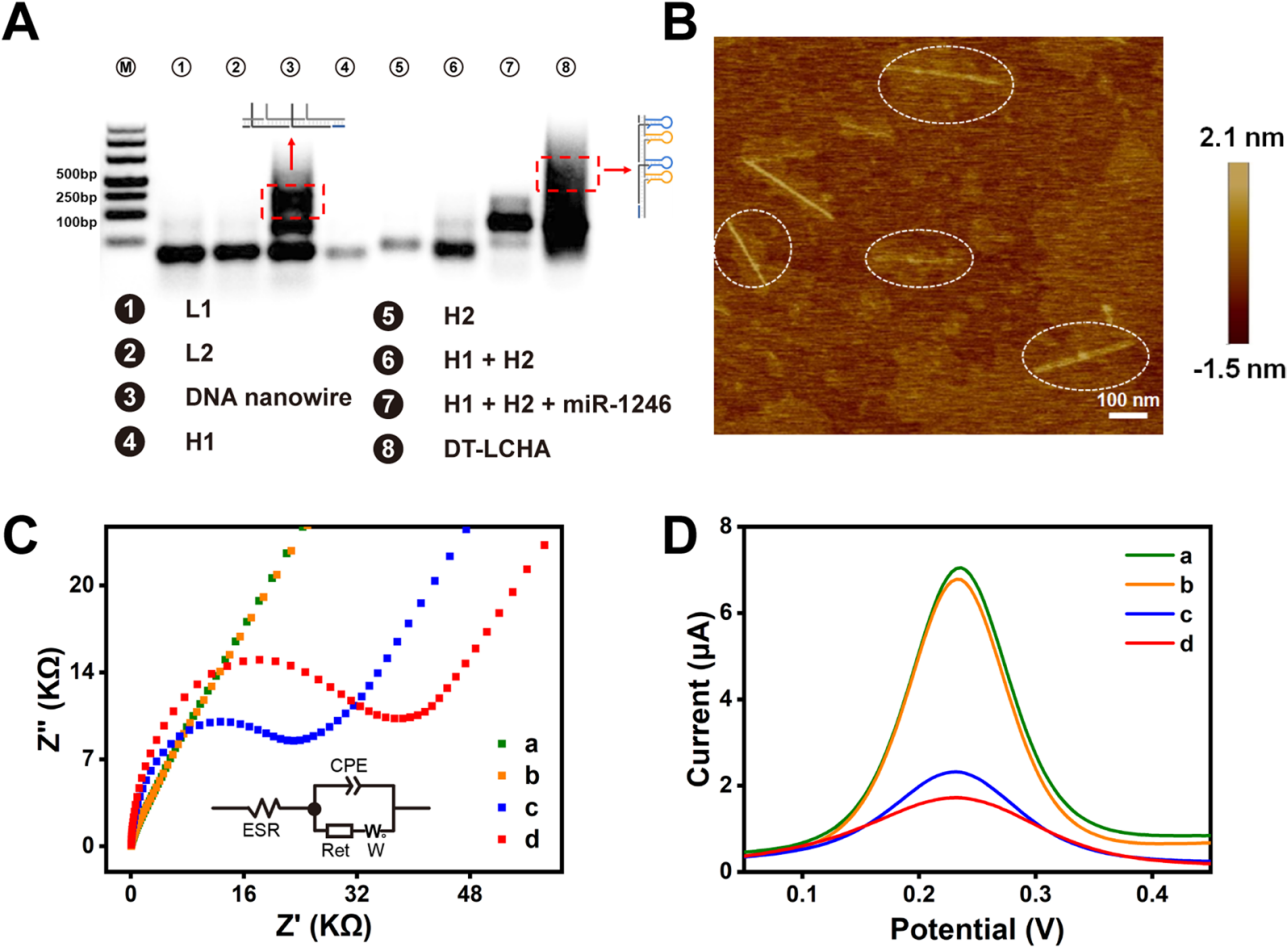
**A** Agarose gel electrophoresis analysis of DT-LCHA, lanes from left to right: Marker; L1; L2; L1 + L2 + Trigger; H1; H2; H1 + H2; H1 + H2 + miR-1246; Nanowire + H1 + H2 + miR-1246. **B** Atom Force Microscope imaging of DT-LCHA. Characterization of the electrochemical biosensor by EIS (**C**) and SWV (**D**) analysis of bare gold (a); capture probe modification (b); MCH closure (c); DT-LCHA captured by capture probe (d)

To verify the assembly of the DT-LCHA complexes, we performed characterization studies using agarose gel electrophoresis. As illustrated in Fig 1A, L1 (lane 1), L2 (lane 2), H1 (lane 4), and H2 (lane 5) were synthesized as designed, with only one clear band and without any spontaneous reaction. The band in lane 3 appeared much higher, indicating the assembly of L1 and L2 via hybridization chain reaction to form the double-stranded DNA nanowire. When the nanowire was mixed with H1 and H2, a significant upward shift of the band was observed (as seen in lane 8), indicating the successful assembly of the DT-LCHA complex. Moreover, the band in lane 7 showed that the CHA system could be successfully triggered by miRNA-1246, with little background signal. Atomic force microscopy (AFM) was used to further confirm the assembly and morphology of the DT-LCHA. The AFM images showed a scaffold structure on which linear structures with a length of ~400 nm were uniformly dispersed (Fig 1B), which provided compelling evidence of the assembly of the DT-LCHA complex.

The immobilization of the DT-LCHA complex on the electrode surface was verified using electrochemical impedance spectroscopy (EIS), cyclic voltammetry (CV), and square wave voltammetry (SWV). In the EIS plots (Fig 1C), the diameter of the semicircle in the plot corresponds to the transfer resistance (Ret). The curve for the bare gold electrode is almost a straight line, indicating good conductivity and low Ret (curve a). After functionalization of the gold electrode surface with capture probes *via* Au–S interaction, the EIS curve showed a slight shift, indicating successful immobilization of the capture probes onto the gold electrode (curve b). According to the formular of Steel *et al.* that G_DNA_ = G_0_ (z/m) N_A_, where G_DNA_ is the probe surface density in molecules per cm^2^, m is the number of bases in probe DNA, z is the charge of RuHex and N_A_ is the Avogadro’s number [39–41]. The amount of capture probe on the electrode surface was calculate as 4.39 × 10^12^ molecule/cm^2^. After immobilization of 6-mercaptohexanol (MCH) onto the remaining empty binding sites on the electrode surface, an increase of the Ret was observed (curve c). This could be due to the increase in the number of molecules on the electrode surface. After all the empty binding sites were closed using MCH, the immobilization of DT-LCHA complexes onto the electrode surface was carried out; as shown in curve d, addition of 100 nM of the target molecule, resulted in a significant increase in the Ret. This means that the DT-LCHA was successfully triggered by the target molecule at the electrode surface and the resultant complex was successfully captured by the capture probe. The kinetic parameters and equivalent circuit of EIS analysis were calculated and extracted according to the methods of Anita *et al*. [42, 43]. The equivalent circuit can fit the EIS plot well (Additional file 1: Fig S4). The SWV and CV tests showed the same gradual assembly of the DT-LCHA complex on the electrode surface, which correlated well with the EIS conclusions (Fig 1D and Additional file 1: Fig S5). These results fully illustrate the successful assembly and feasibility of the proposed DT-LCHA biosensor.

### Triple signal amplification in DT-LCHA

**Fig. 2:**
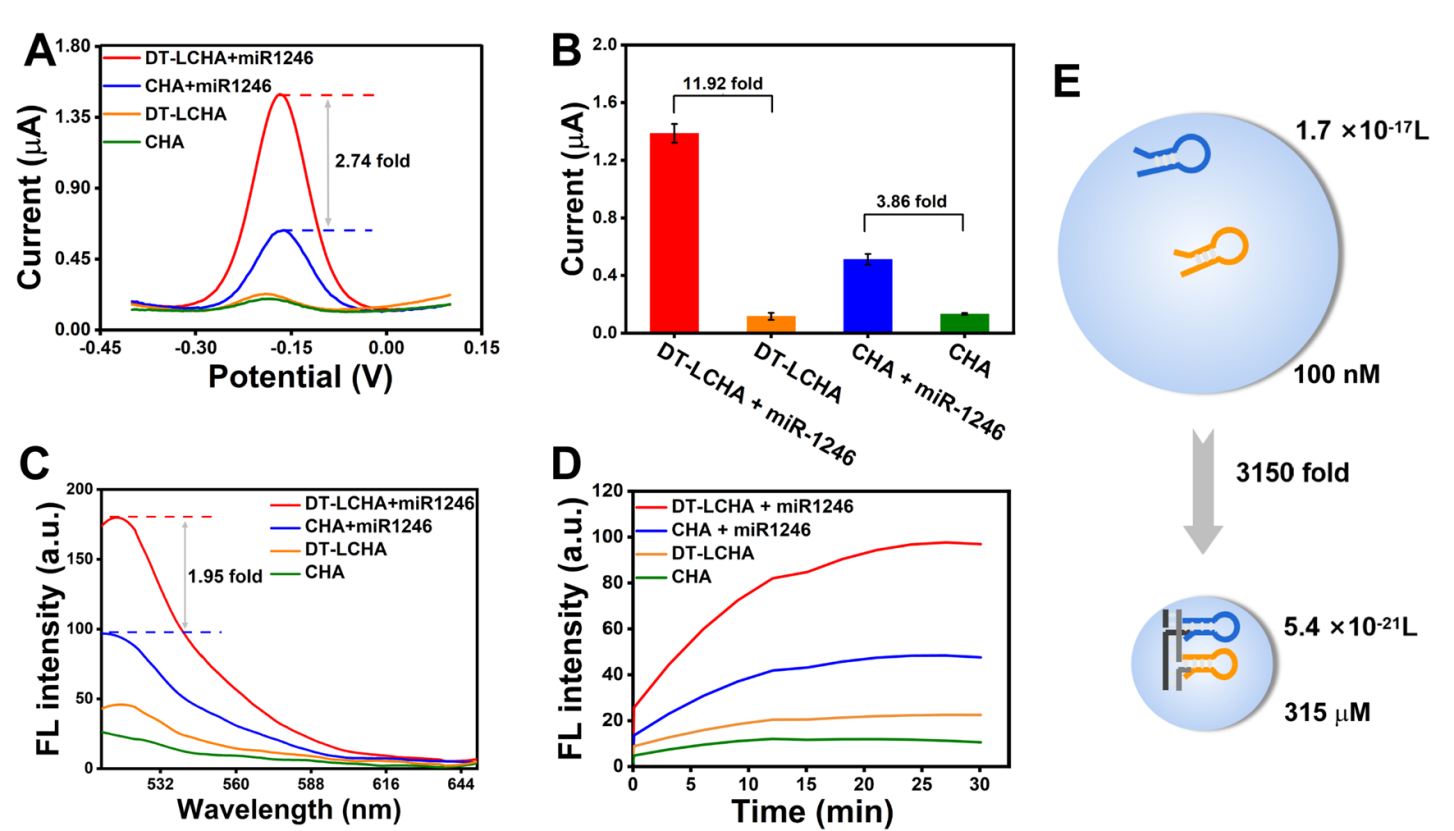
**A** Comparison of the DPV signal of DT-LCHA and conventional CHA. **B** Signal-to-noise ratio of DT-LCHA and conventional CHA. **C** Fluorescence analysis of DT-LCHA. **D** Fluorescence kinetic analysis of DT-LCHA. **E** Comparison of the reaction space and local concentration of traditional CHA and DT-LCHA

To investigate the advantages of the DT-LCHA system, the difference in the electrochemical signal intensity between the DT-LCHA and conventional CHA was assessed using the differential pulse voltammetry (DPV) technique. With the addition of miR-1246, the DT-LCHA emitted strong electrochemical signals with a 2.74-fold signal enhancement compared to conventional CHA (Fig. 2A). The signal-to-noise ratio was greatly improved in the DT-LCHA, with a 11.92-fold improvement as compared to a 3.86-fold improvement in conventional CHA (Fig. 2B). These results demonstrate that the DT-LCHA has a great detection range and facilitates the detection of miRNA targets even when they are present in a low concentration. Fluorescence spectroscopy was used to further reveal the advantages of this localized strategy. The DNA hairpin H1, modified with the fluorophore FAM and the quencher BHQ1 at opposite sites, can emit fluorescence when triggered by the target. As shown in Fig. 2C, signals produced in the DT-LCHA were 1.95 times more amplified than those in conventional CHA. These results demonstrate the enhanced amplification of signals in the DT-LCHA. In addition, kinetic monitoring of fluorescence further verified the enhanced amplification of signals in the DT-LCHA approach. The fluorescence signal strength in DT-LCHA peaks in about 20 min, and it is almost 2 times higher than that in conventional CHA (Fig. 2D). This proves that the DT-LCHA has good efficiency. Interestingly, the electrochemical platform based on the DT-LCHA shows higher signal enhancement than the fluorescent platform. This is owing to the fact that in the electrochemical approach, only a small number of CHA circuits need triggering while the rest occur spontaneously as a cascade, and the whole DT-LCHA complex can be captured by the electrode.

To explain the signal enhancement effect of localization, we used the collision theory equation V = 1/cN (where c is the hairpin concentration and N represents Avogadro’s constant). The collision volume of traditional CHA by using collision theory,

$${\text{V}}_{{{\text{CHA}}}} = { 1 }/{\text{ c}}_{{{\text{CHA}}}} {\text{N}}$$

where c_CHA_ characterizes the concentration of hairpins 100 nM, and N is Avogadro’s constant 6.02 × 10^23^. The volume of traditional CHA was 1.7 × 10^–17^ L. Then the calculation the volume of DT-LCHA by using sphere calculation formula,

$${\text{V}}_{{{\text{DT}} - {\text{LCHA}}}} = { 4 }/{ 3 } \times \, \pi {\text{R}}^{{3}}$$

where R represent the distance between H1 and H2 on the DNA nanowire, where the distance between two hairpins is decreased to 32 base pairs (10.88 nm). The volume of DT-LCHA was 5.4 × 10^–21^ L. Then calculating the collision concentration of localized CHA by using collision theory,

$${\text{c}}_{{{\text{DT}} - {\text{LCHA}}}} = { 1 }/{\text{ V}}_{{{\text{DT}} - {\text{LCHA}}}} {\text{N}}$$

where V characterizes the volume of sphere volume of DT-LCHA. The concentration of CHA localized on DNA nanowire was 315 M, which is 3150 times higher than that observed in traditional CHA (Fig. 2E). This shows that there is an improvement in collision frequency, which results in higher reaction speed and efficiency.

### Analytical performance of the biosensor

**Fig. 3:**
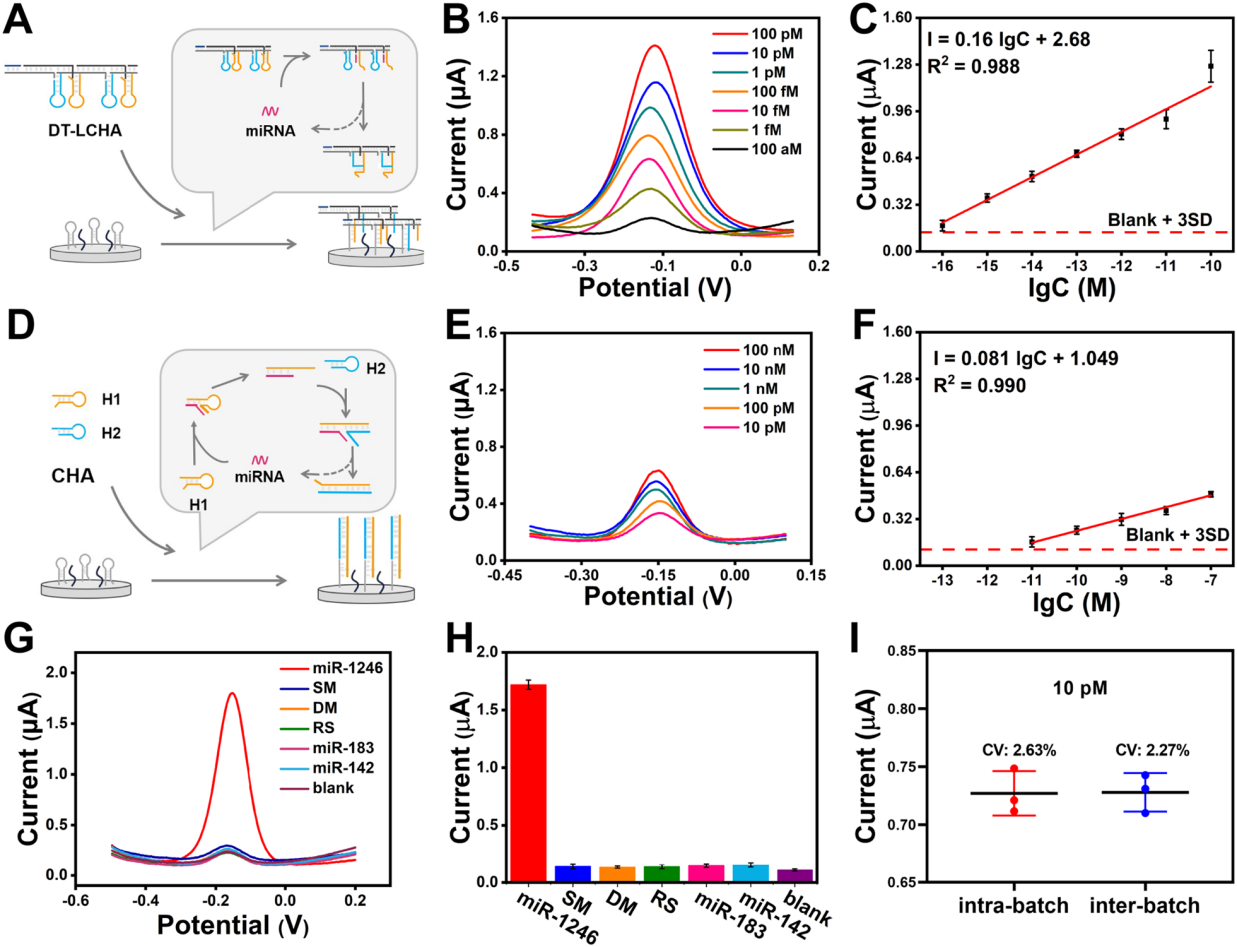
Analytical performance of the biosensor. **A** Schematic illustration of the DT-LCHA strategy. **B** DPV responses and (**C**) corresponding calibration curve of target miRNA with the concentration from 100 aM to 100 pM using DT-LCHA. **D** Schematic illustration of the tradition CHA strategy. **B** DPV responses and (**C**) corresponding calibration curve of target miRNA with the concentration from 10 pM to 100 nM using DT-LCHA. **G** DPV signal and (**H**) DPV results of target miRNA and five interferences. **I** Intra-batch inter-batch variation of DT-LCHA. The error bars are the standard deviations by three repetitive assays (n = 3)

After the optimization of the reaction temperature and time (Additional file 1: Figs S6, S7), the sensitivity of the biosensor was studied using different concentrations of sEV-miR-1246. To demonstrate the superior sensitivity of the developed DT-LCHA biosensor, we conducted DPV analysis on both the DT-LCHA (Fig. 3A) and conventional CHA (Fig. 3D). As illustrated in Fig. 3B, the DT-LCHA exhibited a higher signal output than conventional CHA (Fig. 3E) at the same target concentration. Furthermore, the DT-LCHA demonstrated a broader linear range between 100 aM and 100 pM. The linear regression equation of the DT-LCHA was I = 0.16 lgC + 2.68 (R^2^ = 0.988) (Fig. 3C). Using the blank + 3SD rule, the limit of detection of the DT-LCHA was found to be as low as 24.55 aM, 6213.43 times lower than that of conventional CHA (Fig. 3F). These results provide comprehensive evidence of high sensitivity in the DT-LCHA strategy. The superior sensitivity of the developed DT-LCHA biosensor may be related to the design of triple signal amplification in DT-LCHA. Not only does the localization effect leads to an increase in the number of CHA hybridization chains triggered per unit time, but also the increase in the number of DNA scaffolds captured on the electrode can also lead to an increase in RuHex adsorption, resulting in stronger signal output. Furthermore, only a small amount of CHA needs to be triggered under the condition of extremely low concentration of target miRNA, that is, DT-LCHA is captured on the electrode, and more RuHex is adsorbed under the action of nanowire scaffold, resulting in the enhancement of electrochemical signal. These results provide comprehensive evidence of high sensitivity in the DT-LCHA strategy.

For ascertaining the specificity of the DT-LCHA-based biosensor, a comprehensive analysis involving single-base mismatches, double-base mismatches, random sequences, and two homologous sEV-miRNAs (sEV-miR-183 and sEV-miR-142) was conducted using DPV tests. The results, as depicted in Fig. 3G, H, unequivocally demonstrate an 11-fold higher signal response for fully paired targets in comparison to single-base mutations. This prominent disparity serves as a testament to the excellent specificity of the proposed platform. The excellent specificity of proposed platform due to the proper design of DNA hairpins, especially the proper distribution of toehold length and double-stranded hybridized part in CHA hairpin. Additionally, the reproducibility of the biosensor was rigorously evaluated by analyzing miRNA at a concentration of 10 pM, considering both intrabatch and interbatch variability. The obtained data, presented in Fig. 3I, reveal a variable coefficient of approximately 3%, thereby indicating the platform’s commendable reproducibility. The commendable reproducibility performance is attributed to the high yield of DT-LCHA prepared by six designed DNA strands, which brings unparalleled stability and uniformity.

In comparison to alternative biosensors employed in the detection of sEV-miRNA (Additional file 1: Table S2), the newly devised DT-LCHA-based platform exhibits superior sensitivity and specificity. While the surface-enhanced Raman scattering (SERS)-based approach demonstrates heightened sensitivity, the intricate operational requirements and costly infrastructure associated with it may impose restrictions on its clinical feasibility. Conversely, the DT-LCHA-based electrochemical biosensor showcases noteworthy advantages such as economical implementation, rapid detection time, and user-friendly operation, thereby exhibiting substantial potential for facilitating point-of-care detection applications.

### Determination of cell-line-derived sEV-miRNA

**Fig. 4:**
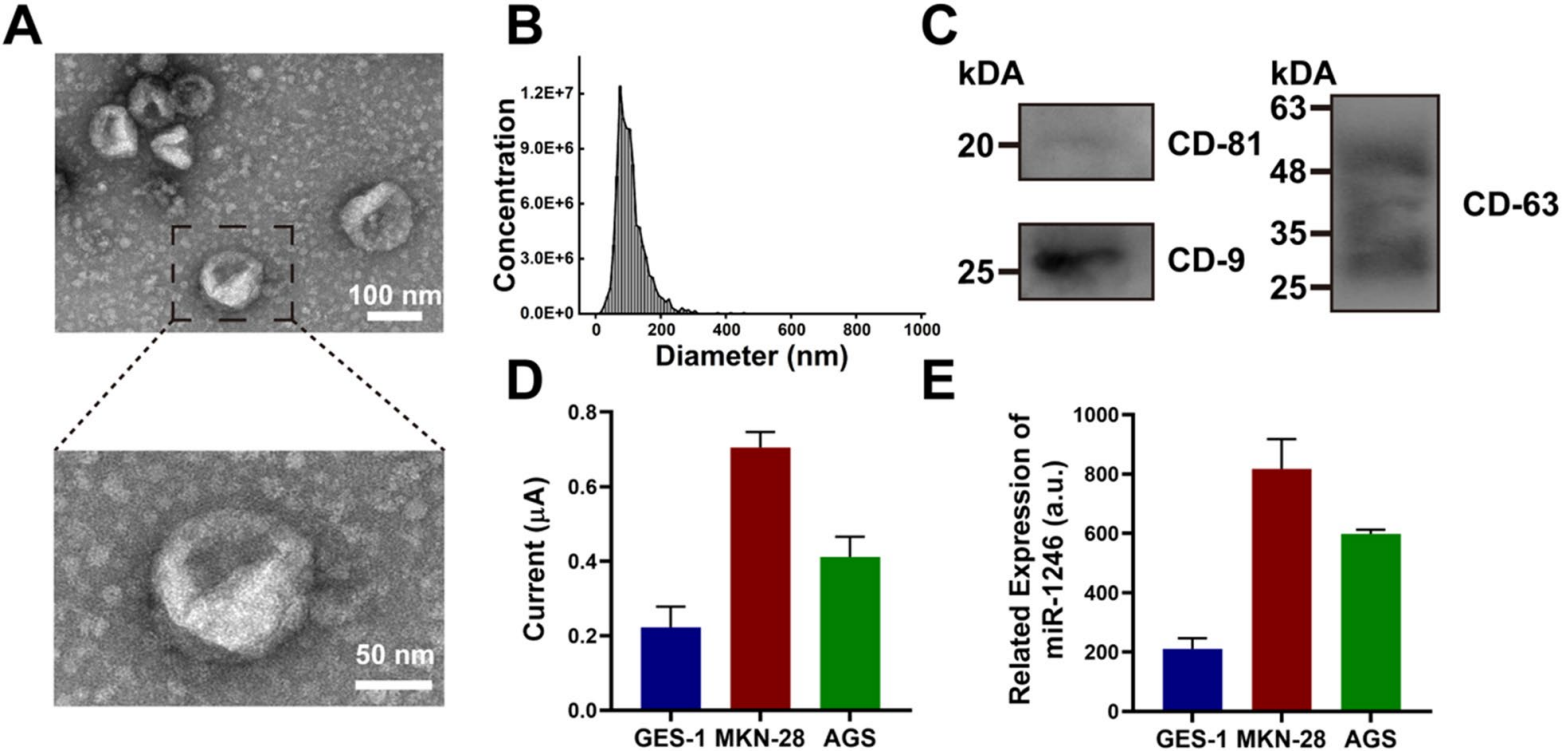
**A** TEM images of sEV extracted from the model cell line GES-1 (upper scale: 100 nm, lower scale: 50 nm). **B** NTA analysis of sEV from GES-1. **C** Western blot analysis of sEV marker proteins CD-81, CD-9 and CD-63. **D** DPV signal of extracted sEV from three model cell lines GES-1, MKN-28 and AGS. **E** RT-qPCR analysis of extracted sEV from three model cell lines GES-1, MKN-28 and AGS. The error bars are the standard deviations by three repetitive assays (n = 3)

Prior to the determination of sEV-miRNA, we used the ultracentrifugation method to extract sEVs from a gastric epithelial cell line (GES-1). The sEVs isolated from the GES-1 cell line were characterized using standard protocols. The ultrastructure of sEVs under a transmission electron microscope revealed a typical disk shape with a diameter of 110 nm, which was consistent with the results of the nanoparticle tracking analysis (NTA) (Fig. 4A, B). The specific marker proteins of sEVs, including CD-81, CD-9, and CD-63, were characterized by using western blotting (Fig. 4C). The findings demonstrate that the extracted sEVs meet the definition and exhibit high purity.

Subsequently, we selected a total of three cell lines—two gastric cancer cell lines and a normal gastric epithelial cell line. After extracting total RNA, we used the proposed platform and RT-qPCR to quantify sEV-miR-1246. As shown in Fig. 4D, DPV analysis showed the sEV-miR-1246 expression in the gastric tumor cell lines was higher than that in the normal gastric cell line, with MKN-28 sEVs exhibiting the highest miR-1246 expression. The proposed platform exhibited a good correlation with RT-qPCR analysis for sEV-miR-1246 (Fig. 4E). These results confirm the high accuracy of the electrochemical biosensor based on the DT-LCHA.

### Clinical sample analysis on the DT-LCHA platform

**Fig. 5:**
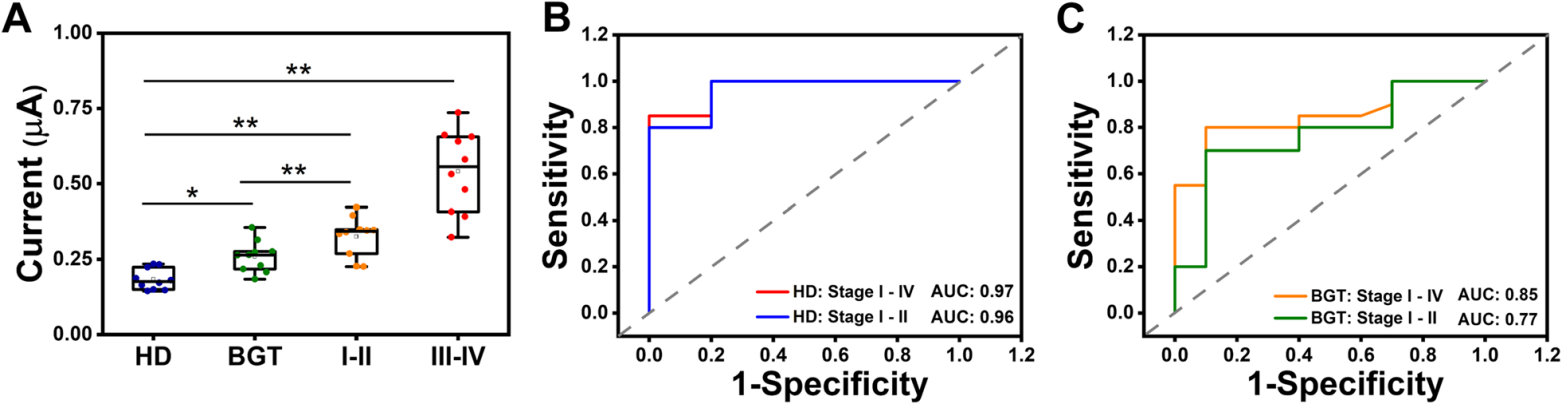
Clinical sample analysis on DT-LCHA platform. **A** The electrochemical signal for sEV-miR-1246 profiling of Healthy doners (HD); benign gastric tumor patients (BGT); Stage I-II gastric cancer patients (Stage I-II); Stage III-IV gastric cancer patients (Stage III-IV) (*p < 0.05, **p < 0.01; paired t test). **B** ROC curve between gastric cancer patients (Stage I-IV, Stage I-II) and healthy donors. **C** The ROC curve was between gastric cancer patients (Stage I-IV, Stage I-II) and benign gastric tumor patients

To assess the application potential of the proposed platform, a cohort of 40 patients (n = 40) was recruited, comprising 10 healthy donors, 10 patients with benign gastric tumors, 10 patients with gastric cancer in stages I–II, and 10 patients with gastric cancer in stages III–IV (Additional file 1: Table S3). The DPV signals of the cohort, depicted in Fig. 5A, revealed a substantially elevated current for sEV-miR-1246 in gastric cancer patients, with an apparent trend of increasing levels as the disease progressed. Furthermore, the proposed platform effectively differentiated between the levels of sEV-miR-1246 in patients with gastric cancer and those in patients with benign gastric tumors. To determine the diagnostic performance, receiver operating characteristic (ROC) analysis was conducted, comparing gastric cancer patients with healthy donors, yielding an impressive area under the curve (AUC) of 0.97 (all stages; CI 0.873–1; sensitivity: 85%; specificity: 100%) and 0.96 (stage I–II; CI 0.883–1; sensitivity: 100%; specificity: 80%), approaching the ideal value of 1. Moreover, diagnostic performance was evaluated within the cohort of benign gastric tumor patients and gastric cancer patients by using the proposed platform. ROC analysis revealed the favorable diagnostic capability of the proposed platform for gastric cancer across all stages, achieving a sensitivity of 80% and a specificity of 90% (AUC: 0.85; CI 0.688–1). Additionally, it demonstrated promising performance in predicting stage I–II gastric cancer in patients and benign gastric tumor in patients, yielding a sensitivity of 70% and specificity of 90% (AUC: 0.77; CI 0.553–0.987) (Fig. 5C). The cut off value of GC patients was calculated as 0.251 μA (CI 0.873 to 1; sensitivity of 85%, specificity of 100%) (Additional file 1: Fig S8) according to the Youden index. Collectively, these results show the significant clinical detection potential of the proposed platform.

## Conclusion

A novel electrochemical biosensing platform that used the DT-LCHA was developed for rapid and sensitive detection of sEV-miRNA. In addition to its accurate response capabilities, the DT-LCHA offers several key advantages. Leveraging the innovative concept of substrate transduction from dispersion to localization, the DT-LCHA exhibits remarkable sensitivity in detecting target miRNA. This biosensing platform demonstrates the ability to detect sEV-miRNA at concentrations as low as 24.55 aM within a 20 min timeframe. The simplicity of DT-LCHA preparation is another notable feature, as it involves the straightforward binding of CHA hairpins to a single DNA nanowire. Furthermore, the clinical potential of this platform was validated by successfully detecting sEV-miR-1246 in gastric cancer patients, enabling the identification of individuals with early-stage gastric cancer. Overall, our results suggest that the DT-LCHA is a promising electrochemical biosensing platform with high sensitivity, specificity, and reproducibility and has potential for widespread clinical use.

### Supplementary Information

**Additional file 1: Figure S1.** Overview of the proposed platform based on DT-LCHA. **Figure S2.** Design details of DT-LCHA. (A) The design of the DNA Nanowire. (B) The design of the reaction progress of DT-LCHA. **Figure S3.** Secondary structure and thermodynamic analysis of DT-LCHA by NUPACK Software[1]. (https://alpha.nupack.org). **Figure S4.** EIS equivalent circuit and kinetic parameters and charge resistance transfer. **Figure S5.** Characterization of the electrochemical biosensor by CV analysis of bare gold (a); capture probe modification (b); MCH closure (c); DT-LCHA captured by capture probe (d). **Figure S6.** The effect of the reaction temperature of DT-LCHA. **Figure S7.** The effect of the reaction time of DT-LCHA. **Figure S8.** Signal cut off of GC diagnostic. **Table S1.** DNA sequences of used in this assay. **Table S2**. Comparison of different biosensors for detecting sEV-miRNA. **Table S3.** Clinical information for healthy donors (HD), benign gastric tumor patients (BGT) and gastric cancer patients (GC).

## Data Availability

Not applicable.
