# Supplementary material for: A sensitive and rapid electrochemical biosensor for sEV-miRNA detection based on domino-type localized catalytic hairpin assembly
Source: J Nanobiotechnology. 2023 Sep 9;21:328. doi: 10.1186/s12951-023-02092-x (PMC10492399; doi:10.1186/s12951-023-02092-x)
Supplement: Supplementary file 1 — Additional file 1: Figure S1. Overview of the proposed platform based on DT-LCHA. Figure S2. Design details of DT-LCHA. (A) The design of the DNA Nanowire. (B) The design of the reaction progress of DT-LCHA. Figure S3. Secondary structure and thermodynamic analysis of DT-LCHA by NUPACK Software[1]. (https://alpha.nupack.org). Figure S4. EIS equivalent circuit and kinetic parameters and charge resistance transfer. Figure S5. Characterization of the electrochemical biosensor by CV analysis of bare gold (a); capture probe modification (b); MCH closure (c); DT-LCHA captured by capture probe (d). Figure S6. The effect of the reaction temperature of DT-LCHA. Figure S7. The effect of the reaction time of DT-LCHA. Figure S8. Signal cut off of GC diagnostic. Table S1. DNA sequences of used in this assay. Table S2. Comparison of different biosensors for detecting sEV-miRNA. Table S3. Clinical information for healthy donors (HD), benign gastric tumor patients (BGT) and gastric cancer patients (GC). [file 12951_2023_2092_MOESM1_ESM.docx]

**Supporting information**

**A sensitive and rapid electrochemical biosensor for sEV-miRNA detection based on domino-type localized catalytic hairpin assembly**

*Wenbin Li^12‡^, Wen Wang^123‡^, Shihua Luo^4‡^, Siting Chen^12^, Tingting Ji^12^, Ningcen Li^12^, Weilun Pan^12^, Xiaohe Zhang^12^, Xiaojing Wang^1^, Ke Li^1^, Ye Zhang^12^*, Xiaohui Yan^12^*.*

^1^Laboratory Medicine Center, Department of Laboratory Medicine, Nanfang Hospital, School of Basic Medical Sciences, Southern Medical University, Guangzhou 510515, P.R. China

^2^Guangdong Engineering and Technology Research Center for Rapid Diagnostic Biosensors, Nanfang Hospital, Southern Medical University, Guangzhou 510515, P.R. China

^3^Medical Laboratory of the Third Affiliated Hospital of Shenzhen University, Shenzhen 518001, P.R. China

^4^Center for Clinical Laboratory Diagnosis and Research, The Affiliated Hospital of Youjiang Medical University for Nationalities, Baise 533000, Guangxi, PR China

**Keywords:** DNA nanowire, catalyzed hairpin assembly, sEV-miRNAs, early cancer diagnostics

*Corresponding author at: Laboratory Medicine Center, Department of Laboratory Medicine, Nanfang Hospital, School of Basic Medical Sciences, Southern Medical University, Guangzhou 510515, P.R. China. E-mail address: gzyanxh@smu.edu.cn (Xiaohui Yan); Zhangye232@i.smu.edu.cn (Ye Zhang).

^‡^ These authors contributed equally to this work.


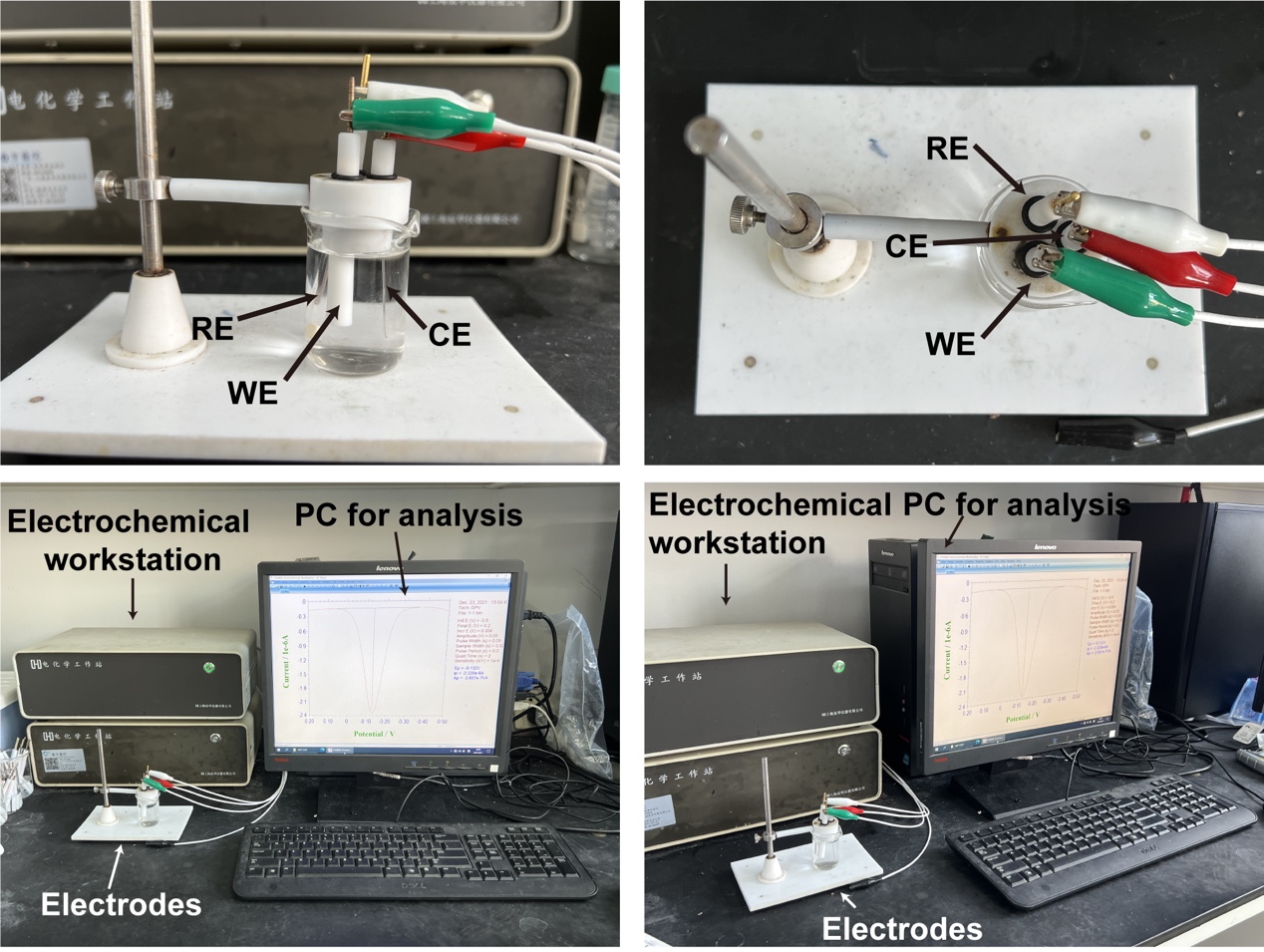


Figure S1. Overview of the proposed platform based on DT-LCHA.


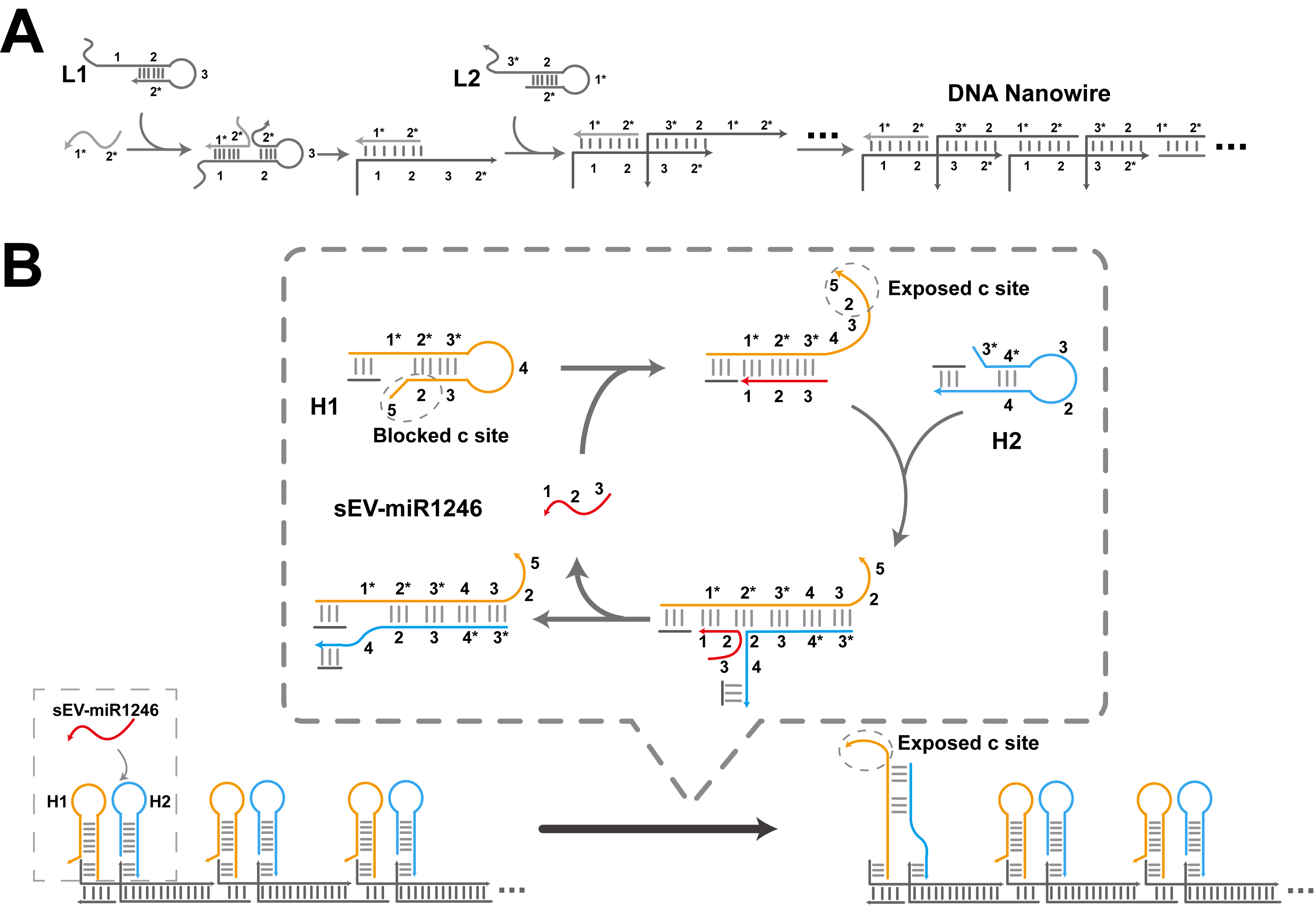


Figure S2. Design details of DT-LCHA. (A) The design of the DNA Nanowire. (B) The design of the reaction progress of DT-LCHA.


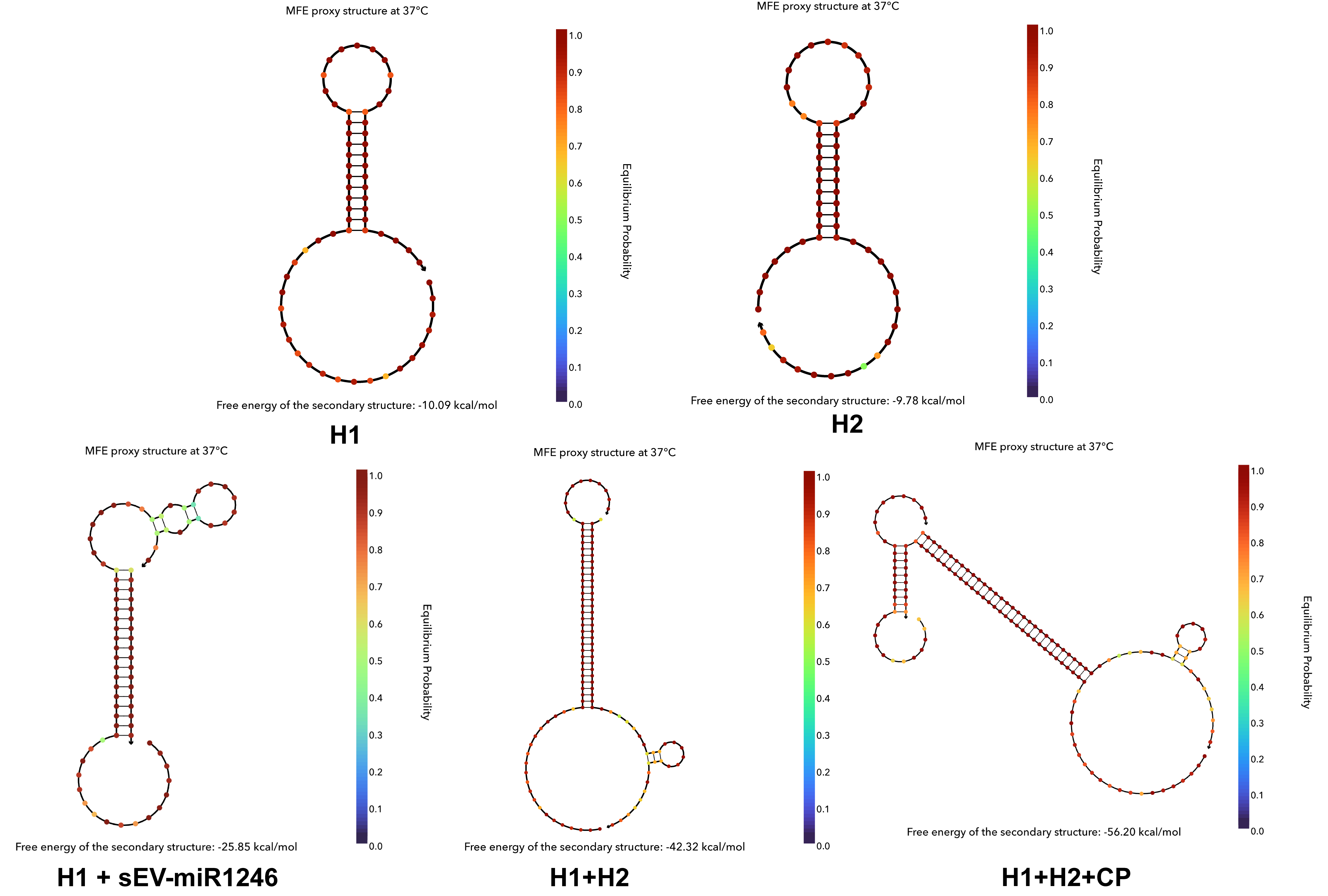


Figure S3. Secondary structure and thermodynamic analysis of DT-LCHA by NUPACK Software[1]. (<https://alpha.nupack.org>)


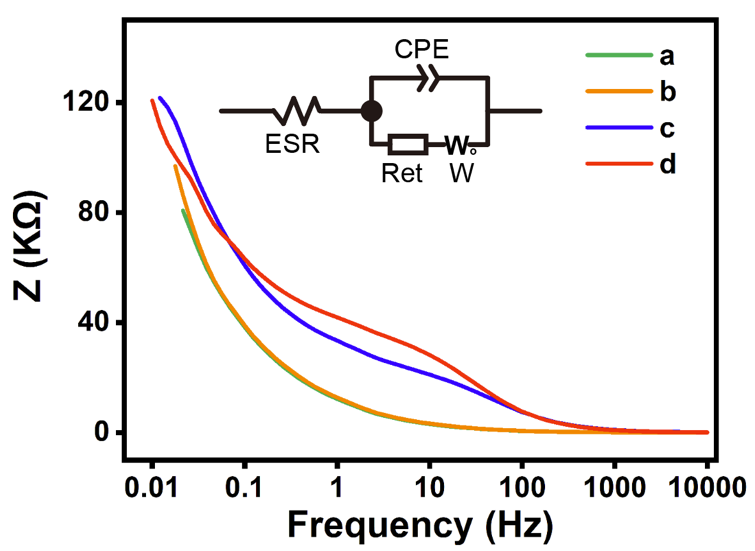


Figure S4. EIS equivalent circuit and kinetic parameters and charge resistance transfer.


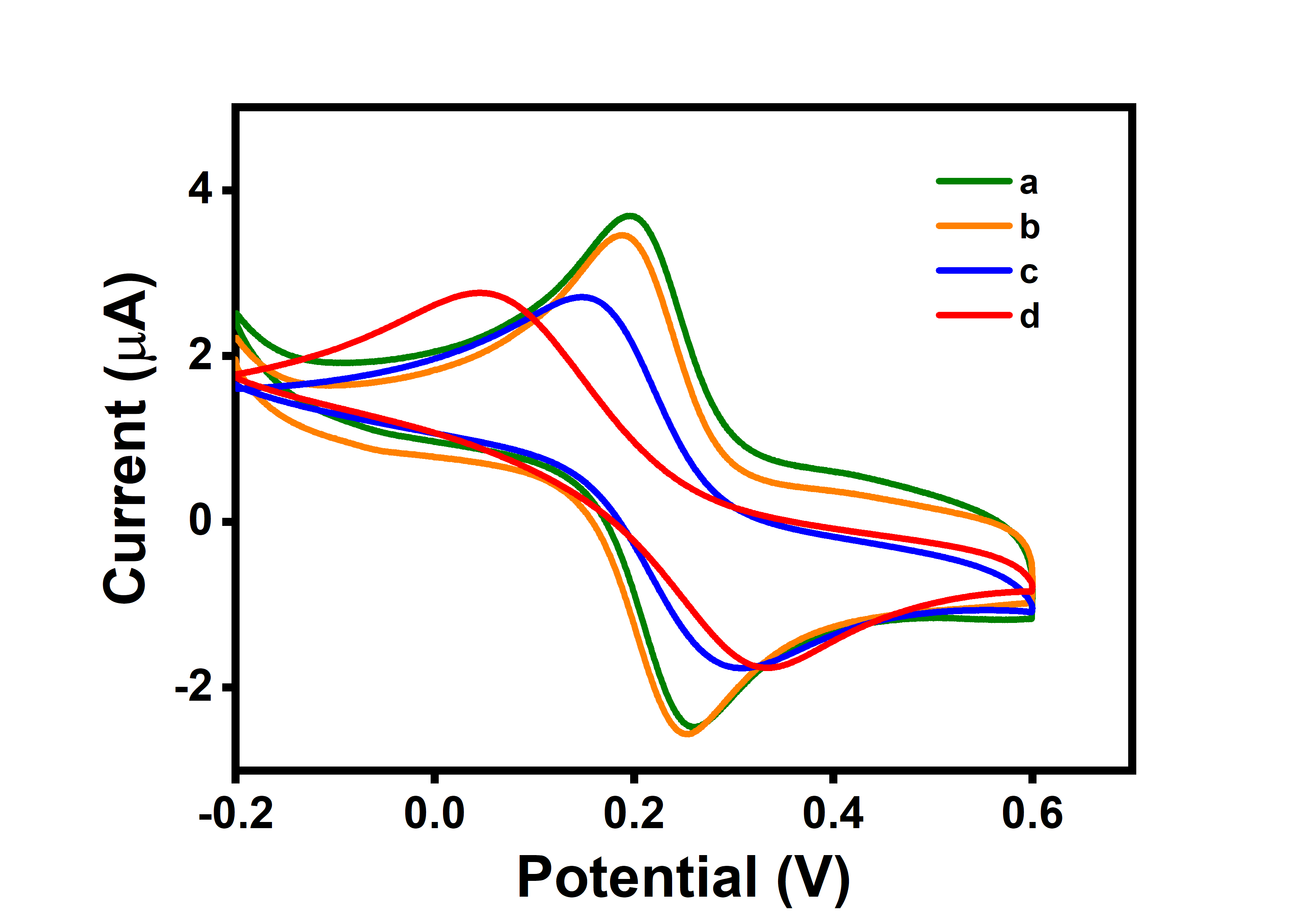


Figure S5. Characterization of the electrochemical biosensor by CV analysis of bare gold (a); capture probe modification (b); MCH closure (c); DT-LCHA captured by capture probe (d).


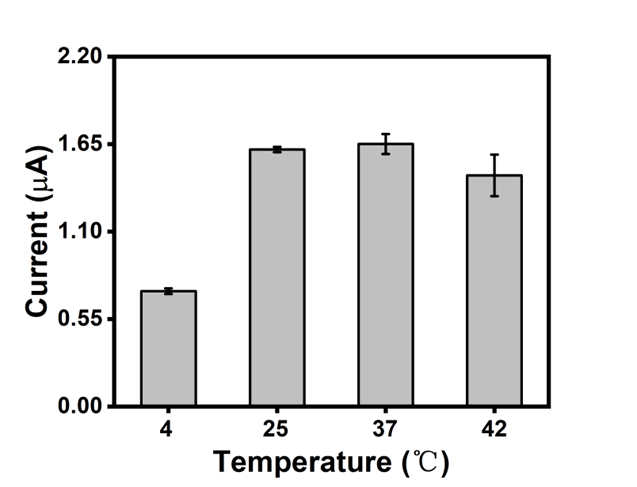


Figure S6. The effect of the reaction temperature of DT-LCHA.


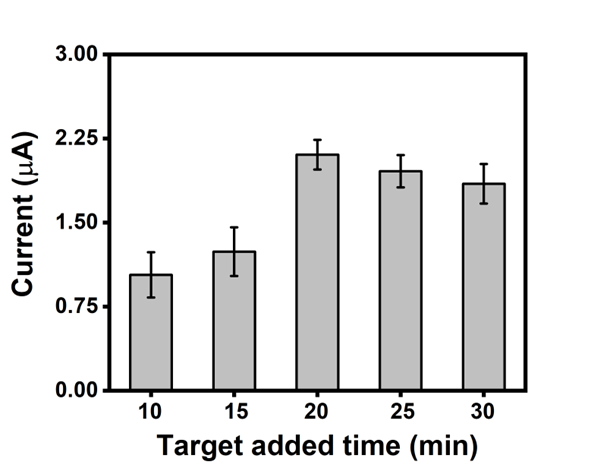


Figure S7. The effect of the reaction time of DT-LCHA.


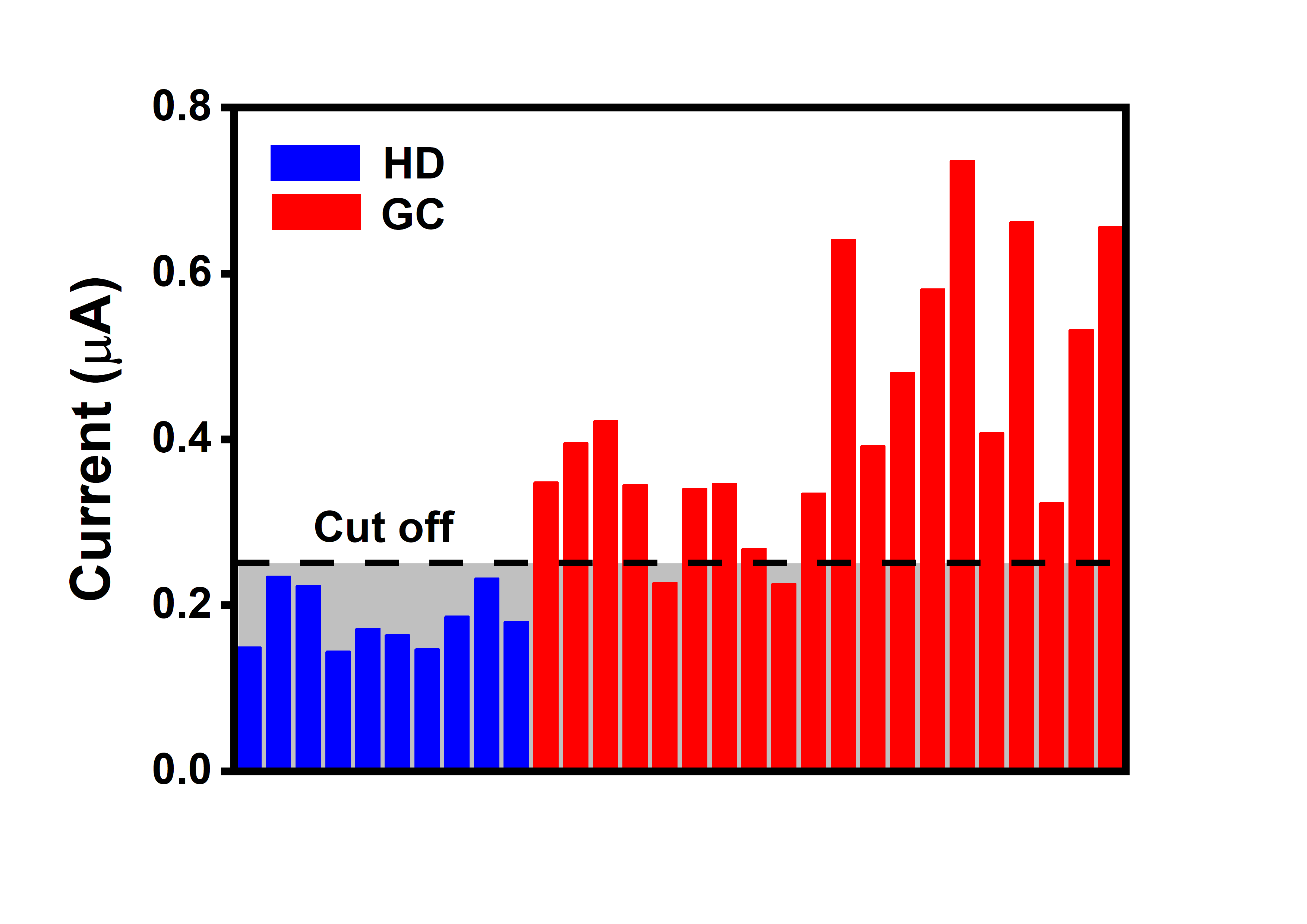


Figure S8. Signal cut off of GC diagnostic.

Table S1. DNA sequences of used in this assay

| **Name** | **Sequence** |
| --- | --- |
| Capture probe (CP) | GCTACGCACTCAGTAGCAAAAACGATGCCCCC-SH(CH_2_)_6_ |
| H1 | CCCACACGTCAAACCTTCCTGCCCAAAAATCCATTGATGTACTAGGAATGGATTTTTGCTAC |
| H2 | TCCATTCCTAGTACATCAATGGATTTTTGGGATGTACTAGGTCCTCTCCTGCTACACA |
| miR-1246 | AAUGGAUUUUUGGGCAGG |
| Trigger | AGTCTAGGATTCGTAGCGTGTTGCTTATTGTG |
| L1 | GACAATAAGCAACACGCTACGAATCCTAGACTGACACTAAGTAGTCTAGGATTCGTAGCGTGTTAAGGTTTGACGTGTGGG |
| L2 | TGTGTAGCAGGAGAGGAGTCTAGGATTCGTAGCGTGTTGCTTATTGTGAACACGCTACGAATCCTAGACTACTTAGTGTC |

Table. S2 Comparison of different biosensors for detecting sEV-miRNA

| **Method** | **Technique** | **Reaction Time (min)** | **Detection range** | **LOD** | **Selectivity** | **Ref.** |
| --- | --- | --- | --- | --- | --- | --- |
| RCA with CRISPR/Cas9 | Fluorescence | 180 | 1 pM-10 nM | 90 fM | >1.5 | [2] |
| PER | Electrochemical | Not given | 1 fM-1 nM | 0.29 fM | >2.5 | [3] |
| Bipedal DNA walkers | Electrochemical | 180 | 0.1 fM -0.1 pM | 67 aM | >12 | [4] |
| LSDR | Electrochemical | Not given | 10 fM-70 fM | 2.3 fM | >50 | [5] |
| HCR with DNAzyme | Fluorescence | 120 | 10 pM-10 nM | 10 pM | Not given | [6] |
| HCR with Exo I | Electrochemical | 180 | 0.1 fM -100 nM | 53 aM | >7.5 | [7] |
| AuNPs deposited as nanopillar | SERS | Not given | 1 aM to 100 nM | 1 aM | >5 | [8] |
| L-DCDR | Electrochemical | 120 | 0.1 fM-1nM | 65 aM | Not given | [9] |
| TMSDR | Electrochemical | Not given | 1 fM-1 nM | 2.75 fM | Not given | [10] |
| NG-CHA | Fluorescence | 30 min | 1 pM-50 nM | 0.8 pM | Not given | [11] |
| NL-CHA | Electrochemical | 20 min | 100 aM-100 pM | 24.55 aM | >10 | This work |

Table S3: Clinical information for healthy donors (HD), benign gastric tumor patients (BGT) and gastric cancer patients (GC).

| Index | Age | Stage |
| --- | --- | --- |
| HD 1 | 58 | - |
| HD 2 | 25 | - |
| HD 3 | 17 | - |
| HD 4 | 36 | - |
| HD 5 | 57 | - |
| HD 6 | 24 | - |
| HD 7 | 35 | - |
| HD 8 | 42 | - |
| HD 9 | 32 | - |
| HD 10 | 56 | - |
| BGT 1 | 33 | - |
| BGT 2 | 44 | - |
| BGT 3 | 57 | - |
| BGT 4 | 49 | - |
| BGT 5 | 44 | - |
| BGT 6 | 45 | - |
| BGT 7 | 56 | - |
| BGT 8 | 41 | - |
| BGT 9 | 47 | - |
| BGT 10 | 55 | - |
| GC 1 | 45 | I |
| GC 2 | 49 | I |
| GC 3 | 59 | IV |
| GC 4 | 74 | IV |
| GC 5 | 57 | II |
| GC 6 | 71 | II |
| GC 7 | 64 | II |
| GC 8 | 71 | II |
| GC 9 | 61 | II |
| GC 10 | 52 | I |
| GC 11 | 50 | I |
| GC 12 | 64 | IV |
| GC 13 | 66 | IV |
| GC 14 | 30 | II |
| GC 15 | 64 | III |
| GC 16 | 70 | III |
| GC 17 | 75 | IV |
| GC 18 | 76 | III |
| GC 18 | 57 | III |
| GC 18 | 81 | IV |

Reference

1. Zadeh JN, Steenberg CD, Bois JS, Wolfe BR, Pierce MB, Khan AR, Dirks RM, Pierce NA: **NUPACK: Analysis and design of nucleic acid systems.** *J Comput Chem* 2011, **32:**170-173.

2. Wang R, Zhao X, Chen X, Qiu X, Qing G, Zhang H, Zhang L, Hu X, He Z, Zhong D, et al: **Rolling Circular Amplification (RCA)-Assisted CRISPR/Cas9 Cleavage (RACE) for Highly Specific Detection of Multiple Extracellular Vesicle MicroRNAs.** *Anal Chem* 2020, **92:**2176-2185.

3. Li X, Li X, Li D, Zhao M, Wu H, Shen B, Liu P, Ding S: **Electrochemical biosensor for ultrasensitive exosomal miRNA analysis by cascade primer exchange reaction and MOF@Pt@MOF nanozyme.** *Biosens Bioelectron* 2020, **168:**112554.

4. Zhang J, Wang LL, Hou MF, Xia YK, He WH, Yan A, Weng YP, Zeng LP, Chen JH: **A ratiometric electrochemical biosensor for the exosomal microRNAs detection based on bipedal DNA walkers propelled by locked nucleic acid modified toehold mediate strand displacement reaction.** *Biosens Bioelectron* 2018, **102:**33-40.

5. Luo L, Wang L, Zeng L, Wang Y, Weng Y, Liao Y, Chen T, Xia Y, Zhang J, Chen J: **A ratiometric electrochemical DNA biosensor for detection of exosomal MicroRNA.** *Talanta* 2020, **207:**120298.

6. He D, Hai L, Wang H, Wu R, Li HW: **Enzyme-free quantification of exosomal microRNA by the target-triggered assembly of the polymer DNAzyme nanostructure.** *Analyst* 2018, **143:**813-816.

7. Guo Q, Yu Y, Zhang H, Cai C, Shen Q: **Electrochemical Sensing of Exosomal MicroRNA Based on Hybridization Chain Reaction Signal Amplification with Reduced False-Positive Signals.** *Anal Chem* 2020, **92:**5302-5310.

8. Lee JU, Kim WH, Lee HS, Park KH, Sim SJ: **Quantitative and Specific Detection of Exosomal miRNAs for Accurate Diagnosis of Breast Cancer Using a Surface-Enhanced Raman Scattering Sensor Based on Plasmonic Head-Flocked Gold Nanopillars.** *Small* 2019, **15:**e1804968.

9. Liu P, Qian X, Li X, Fan L, Li X, Cui D, Yan Y: **Enzyme-Free Electrochemical Biosensor Based on Localized DNA Cascade Displacement Reaction and Versatile DNA Nanosheets for Ultrasensitive Detection of Exosomal MicroRNA.** *ACS Appl Mater Interfaces* 2020, **12:**45648-45656.

10. Tang X, Wang Y, Zhou L, Zhang W, Yang S, Yu L, Zhao S, Chang K, Chen M: **Strand displacement-triggered G-quadruplex/rolling circle amplification strategy for the ultra-sensitive electrochemical sensing of exosomal microRNAs.** *Mikrochim Acta* 2020, **187:**172.

11. Zhang Y, Wu Y, Luo S, Yang C, Zhong G, Huang G, Zhang X, Li B, Liu C, Li L, et al: **DNA Nanowire Guided-Catalyzed Hairpin Assembly Nanoprobe for In Situ Profiling of Circulating Extracellular Vesicle-Associated MicroRNAs.** *ACS Sens* 2022, **7:**1075-1085.
